# Feasibility of implementing formal long-distance mentorship for public health physicians: a case study of Association of Public Health Physicians of Nigeria

**DOI:** 10.1186/s12889-021-11942-y

**Published:** 2021-10-15

**Authors:** Uche Shalom Obi, Chinyere Mbachu, Benjamin S. C. Uzochukwu

**Affiliations:** 1 Department of Community Medicine, University of Nigeria Teaching Hospital, Enugu, Nigeria; 2 Institute of Public Health University of Nigeria, Enugu, Nigeria

**Keywords:** Long-distance mentorship, Willingness, Feasibility, Public health physicians

## Abstract

**Background:**

Conflicting schedules and geographic access limit prospects for mutually beneficial relationships between experts and early career professionals. A formal long-distance mentorship program could address these barriers and potentially bridge the gap of traditional face-to-face mentorship. This study was done to determine the feasibility of implementing a formal long-distance mentorship program amongst public health physicians of Nigeria.

**Method:**

A mixed-method study comprising of in-depth interviews and surveys was used to collect information from members of the Association of Public Health Physicians in Nigeria. A total of 134 survey participants were recruited consecutively during an annual scientific meeting of the association. In-depth interviewees were purposively selected to ensure diversity in expertise, experience, and social stratifiers such as age. Quantitative data were analyzed using descriptive and inferential statistics, while qualitative data were analyzed using thematic content analysis.

**Results:**

Public health physicians of Nigeria are willing to participate in a formal Long-Distance Mentorship Program, and four elements of feasibility were highlighted as necessary for implementing the program. Namely i) capacity to coordinate LDMP, ii) technical expertise and individual competence to provide mentorship, iii) financial capacity to implement and sustain LDMP, and iv) demand for mentorship by mentees. There is a consensus that the organizational structure of the National Postgraduate Medical College of Nigeria and West African College of Physicians provide an enabling environment to initiate a LDMP for public health physicians of Nigeria. The vast human resources with various expertise and the annual National conferences can be leveraged upon to champion and administer the program. However, there is a need for an administrative structure and technical expertise to enable proper coordination. More so, the need for demand creation and the financial requirement was considered gaps that need to be filled to be able to ensure feasibility. Bivariate analysis showed a significant relationship between the dependent variable (preferred role- mentor/mentee) and independent variables (age, year of graduation, and the number of years of practice), while the binary logistic regression model showed that physicians are more likely to participate as mentors with each unit increase in the number of years of practice. This further buttressed the need to commence the mentoring process as soon as trainees gain entrance into the program, as mentorship does not just prepare them for excellent public health practice, but also builds their capacity to mentor the younger and upcoming public health physicians.

**Conclusion:**

There are enabling structures to incorporate a formal long-distance mentorship program for public health physicians in Nigeria, and physicians are willing to participate in such a program. However, the feasibility of establishing a successful and sustainable program will require robust coordination, technical expertise, demand creation, and financial commitment at both institutional and college levels.

## Background

There is an integral connection between mentorship and professional career development within the medical profession [1]. Throughout history, mentors have helped to shape the development of their mentees. Socrates mentored Plato, who mentored Aristotle, who mentored Alexander the Great [2]. Mentorship is critical to career success in any field, as various studies acquiesce that professionals who have been mentored have more career satisfaction [3–5]. Literature also made it clear that physicians who have been mentored feel more supported, are often more satisfied, and have a more successful career [6], yet early-career physicians have difficulties connecting with desired mentors and even fewer opportunities for mentorship beyond residency training [7, 8].

Within the field of public health, mentorship has recently been emphasized in many academic programs as the key driver of every successful career. Once past residency, early-career physicians have fewer mentorship opportunities, while they also will be expected to become mentors themselves. The crucial need for long-distance mentorship amongst public health physicians was further re-emphasized in a plenary session in the 2016 annual conference that was held in the Federal Capital Territory. Mentorship was portrayed as fundamental to the program, however, difficulties in finding someone local, with whom a mentee can develop a mutually beneficial relationship persists [5].

The traditional method of mentorship involves one-on-one mentoring in a synchronous environment [9]. One-on-one mentorship, though effective has been met with several limitations, which have also increased over time with the rapid expansion of health institutions [10]. Consequently, characteristic variation in location, conflicting schedules, and geographic barriers have made it increasingly difficult for trainees to find mentors locally. However, the mentoring process can take on a whole new dimension when people with common goals are limited by aforementioned barriers. Such limitations necessitate mentoring from a distance. Long-distance mentorship fills these gaps; it increases access to desired mentors, lowers the cost of physical meetings, and is not limited by space, time, or location.

With the advent of online applications for teaching and learning, long-distance mentorship has been conceptualized as the online or electronic version of mentoring [11]. It involves e-mentoring through the use of synchronous and asynchronous computer-mediated communication as a means for establishing mentor-mentee relationships virtually [9]. It essentially serves the same purposes as traditional mentorship, but technologically facilitates mentoring relationships. With long-distance mentorship, the interaction between mentor and mentee is enabled via various technological applications such as e-mail, instant messaging, audio and audio-visual conferencing, as well as online discussion boards [12].

Public health training in Nigeria is expanding to include more residents in both academic and community settings. As a result, most residents find themselves training far from their envisaged, or chosen role models and desired mentors, separated by time and distance. Conflicting schedules and geographic separation make it impossible for the residents to access these important people face to face [13]. There is a need to overcome the limitations of traditional mentorship; find ways to communicate effectively over distance, and create communication avenues between geographically separated individuals.

Long-distance mentorship overcomes the limitations of traditional mentorship and bridges the gap of traditional face-to-face mentorship with overwhelming effectiveness [14]. It is a common practice in developing countries with varying degrees of success [14–16]. This is of great value but is mostly lacking in our setting. Institutionalizing an organized formal mentorship program to address the dire need for post-graduate professional guidance within the association will bridge this gap. However, the feasibility of such a program and the willingness of physicians to participate are yet to be ascertained. This work aims to generate context-specific evidence for the feasibility of establishing a formal long-distant mentorship program for public health physicians in Nigeria and to ascertain the willingness of members to participate in such a program.

## Methods

### Study design

This study used a cross-sectional design, with the convergent mixed-method model to explore context-specific evidence for the feasibility of establishing a formal long-distant mentorship program for public health physicians in Nigeria. Qualitative and quantitative data were collected concurrently, and data integrated by merging of findings. The convergent mixed method of data collection was used to enable data triangulation and more robust synthesis of findings. In-depth interviews were used to explore i) stakeholder’s perception of a formal LDMP, ii) feasibility of jumpstarting such program, iii) available resources and gaps, iv) enabling and constraining factors for implementing and sustaining a successful long-distance mentorship program. The qualitative survey was used to elicit information on respondent’s perception of mentoring, willingness to participate, preferred role (mentor/mentee), and willingness to embrace best practices for long-distance mentorship.

### Study setting and study population

This study was conducted in Delta State, Nigeria, amongst Public Health Physicians of Nigeria. This group of physicians is brought together under the Association of Public Health Physicians of Nigeria (APHPN), an association the draws its membership from 36 states of Nigeria and the Federal Capital Territory. The Association of Public Health Physicians of Nigeria (APHPN) comprises medical doctors who have been trained or are undergoing residency training in public health/community medicine in Nigeria. They are uniquely trained in both clinical medicine and public health to provide medical diagnosis and patient care; conduct teaching and research, and serve in various capacities in different parastatals to maintain the health of the population.

Public health physicians work in healthcare settings, public health departments, and government agencies, development organizations, as well as academia. Their work is focused on the population rather than the individual; provision of essential public health services to the population. Public health physicians are present in all the states of the federation and are brought together under the national body which holds scientific meetings annually. All physicians who have some form of training in public health or who are practicing public health are eligible to be members of the association. As at the time of the study, APHPN had a membership strength of 2000.

The study population for the survey comprised of public health physicians who were attending an annual national scientific conference which was hosted by the Delta State chapter of the association in 2018. This comprised of training consultants, resident doctors undergoing training in community medicine or undertaking a Master’s program in Public Health, public health practitioners in health ministries, departments, and agencies. Respondents for in-depth interviews comprised of stakeholders and members of the association who were considered knowledgeable based on their input and wealth of experience within the association.

### Sample selection

For the survey, all members of the association who were attending the annual scientific conference were invited to participate in the study, and only those who gave consent were interviewed. Conference attendees who were not members of the association were excluded from participating in the study.

Respondents for the in-depth interview were purposively selected to ensure maximum variability and representation of diverse perspectives with respect to leadership contributions/experience within the association, area of expertise, and social characteristics such as age. Participants were asked to suggest additional people to be interviewed. Ten prospective respondents were contacted for the in-depth interviews (8 initially and 2 via referral), and seven of them were interviewed. Three prospective respondents were not interviewed as data saturation was reached.

### Quantitative data collection and analysis

Information sheets and consent forms were provided to all eligible participants and they were given time to read through and ask for clarifications from the researchers before documentation of consent. A self-administered structured questionnaire was used to collect respondents’ information on their demographic characteristics, perception of mentoring, willingness and readiness to participate in a formal LDMP, and the capacity in which the respondents preferred to participate (their preferred role-mentor /mentee) in the program. The dependent variable is the preferred role of the respondent (mentor /mentee), while the independent variables include i) age, ii) sex, iii) year of graduation, iv) the number of years of practice as a public health physician. Numeric variables were summarized using means and standard deviations. Categorical variables were summarized using frequencies and proportions. To identify the relationship between the sociodemographic characteristics of the respondents and the capacity in which the respondents preferred to participate (preferred mentor /mentee) in the program, cross-tabulation of the dependent variable- preferred role (mentor /mentee) and independent variables (sex, age, year of graduation, number of years of practice) was conducted. Statistical significance of observed associations was set at *p* < 0.05. To ascertain possible predictors of the capacity in which the respondents were willing to participate (mentor/mentee role), binary logistic regression was conducted with significant independent variables.

### Qualitative data collection and analysis

Participants were contacted either physically or via email with a brief description of the study objectives, their roles as participants in the study, and consent forms. Participants had no prior knowledge about the interviewer’s personal goal. In-depth interviews were conducted using semi-structure interview guide to elicit information on feasibility, available resources, and gaps that need to be filled to be able to implement a formal long-distance mentorship program. Five of the interviews were conducted face-to-face in the offices of the respondents, while two were conducted over the telephone. The interviews were conducted in the English language. Reflexive thinking was applied in the conduct of the interviews and reflexive insights fed into subsequent interviews to minimize subjectivity. Field notes were taken and the interviews were audio-recorded with the consent of the participants. Each interview lasted between forty-five minutes to one hour and none of the interviews were repeated.

Preliminary data analysis proceeded simultaneously with data collection, and emerging findings informed deeper inquiries in subsequent interviews. Audio-recorded interviews were transcribed verbatim and transcripts anonymized with pseudonyms. Proper thematic content analysis began with the reading of two transcripts repeatedly to achieve immersion and derive codes by identifying words that capture key concepts. Initial codes were reviewed and sorted into categories based on linkages between key concepts. The themes that represented key issues with the feasibility of long-distance mentoring were developed for initial coding and inductive analysis. The codes include the perception of respondents on i) organizational capacity to coordinate LDMP; ii) availability of technical expertise/individual competence to provide mentorship; iii) financial capacity of the organization to implement and sustain LDMP, and iv) demand for mentorship by mentees.

The research team includes three public health physicians who are well-grounded in health policy and systems research and qualitative methodology; a professor of public health a senior lecturer and a resident doctor, all in the department of community medicine. All members of the research team have significant prior experience in conducting qualitative research. The interview guide was developed and discussed with experts, to ensure that the guide, probes, and prompts were free of pre-conceived opinions of team members on the subject matter of interest. The study findings have been reported in line with the consolidated criteria for reporting qualitative research (COREQ) [17].

## Results

The response rate was 75%. A total of 141 questionnaires were collected, 6 were filtered as a result of incompleteness and, 134 were analyzed. The results are presented below.

Table 1 shows the characteristics of the respondents. The mean age of respondents was 42 years (±8.8), with the ages fairly evenly distributed across 5-year intervals between ≤35 to ≥46. There were 68 (50.7%) males and 66 (49.3%) females. Registrars had the highest distribution, 48(35.5%) followed by Professors/ consultants/ lecturers 44(32.9%).

**Table 1:** Demographic characteristics of the respondents

Variable	Frequency ***n*** = 134	Proportion %
**Age category**		
≤ 35	36	26.9
36–40	31	23.1
41–45	28	20.9
≥ 46	39	29.1
**Mean age**	42 years (±8.8)	
**Sex**		
Male	68	50.7
Female	66	49.3
**The best description of the respondents position**		
Registrar	48	35.8
Senior registrar	30	22.4
Professor /Consultant /Lecturer	44	32.9
MPH students	12	9.0
**Years in practice category**		
≤ 5	65	48.5
6–10	36	26.9
11 & more	33	24.6

**Fig. 1:**
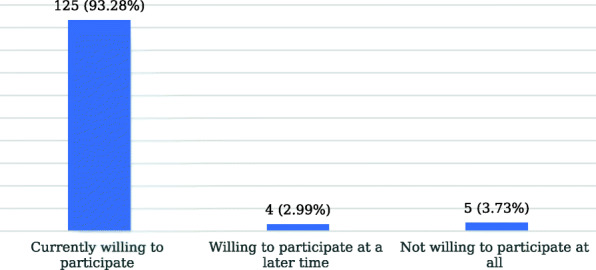
Willingness to participate in a formal LDMP

Figure 1 shows that a total of the 125 (93.28%) respondents were willing to participate in a long-distance mentorship program. Of this category, 109 (87.2%) respondents were also willing to participate in monthly online meetings, while 16 (12.8%) were not willing to participate in monthly online meetings. Of the 16 respondents that indicated a lack of willingness to participate in monthly online meetings: 7 respondents reported that they were busy at the moment and can participate at a later time; 6 respondents noted that monthly meetings are too frequent; 2 respondents indicated that they cannot afford the internet data requirement for monthly online meetings, and 1 respondent said he was already involved in other monthly online meetings.

**Fig. 2:**
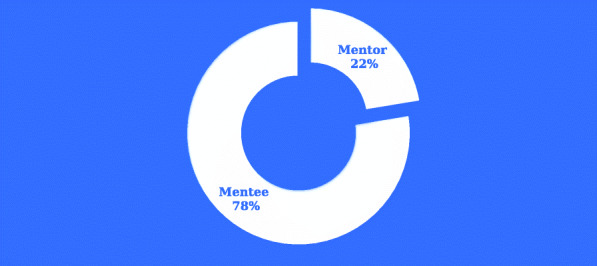
Preferred role of respondents

Figure 2 shows the preferred role of the respondents. Amongst the respondents that were willing to participate in a long-distant mentorship program, 28 (22.4%) would prefer to participate as mentors while 97 (77.6%) would prefer to participate as mentees. All the respondents that chose to participate as mentors were willing to provide all the fundamentals of mentorship to the mentees, while participants that chose to participate as mentees were willing to abide by the fundamentals of mentorship.

### Bivariate analysis

Bivariate analysis showed a significant association between the preferred role (mentor/mentee) and i) age category (Table 2), ii) year of graduation category (Table 3), and iii) the number of years of practice category of the respondents (Table 4). However, there was no significant association between the preferred role and sex of the respondents (Table 5).

**Table 2:** Age category * Respondent’s preferred role in a formal LDMP

		Mentor	Mentee	Total
**Age category**	</=35	0 (0)	36 (100)	36
	36–40	2 (6.5)	29 (93.5)	31
	41–45	10 (35.7)	18 (64.3)	28
	>/=46	17 (43.6)	22 (56.4)	39
	Total	29	105	134

There was a significant association between age category and respondent’s preferred role (*Χ*^2^(1) =28.509, *p* < .001)

**Table 3:** Year of graduation category * Respondent’s preferred role

		Mentor	Mentee	Total
**Year of grad category**	</=2005	15 (22)	53 (77.9)	68
	>/= 2006	14 (21.2)	52 (78.8)	66
	Total	29	105	134

There was a significant association between the year of graduation category and the respondent’s preferred role (*Χ*^2^(1) =19.212, *p* < .001)

**Table 4:** Years in practice category * Respondent’s preferred role in a formal LDMP

		Mentor	Mentee	Total
**Years in practice category**	≤5	2 (3.2)	63 (96.9)	65
	6–10	8 (22.2)	28 (77.7)	36
	≥11	19 (57.6)	14 (42.4)	33
	Total	29	105	134

There was a significant association between years in practice category and respondent’s preferred role (*Χ*^2^(1) =38.345, *p* < .001)

**Table 5:** Sex of respondent * Respondent’s preferred role in a formal LDMP

		Mentor	Mentee	Total
**Sex of respondents**	Male	15 (22%)	53 (77.9)	68
	Female	14 (21.2%)	52 (78.7)	66
	Total	29	105	134

No association was found between sex and preferred role of the respondents (*Χ*^2^(2) > = 0.14, *p* = 0.905)

### Logistic regression analysis

The independent variables (Age, year of graduation, and the number of years of practice) were used to predict the preferred role of respondents (dependent variable). The result of the logistic regression analysis is presented in Table 6 below.

**Table 6:** Binary logistic regression

	B	S.E	Wald	df	Sig.	Exp(B)	Upper	Lower
Age in years	−.060	.077	.612	1	.434	.942	.810	1.095
Year of graduation	.005	.079	.004	1	.949	1.005	.861	1.173
Year of practice	−.130	.057	5.283	1	**.022**	.878	.786	.981
Constant	−4.802	160.612	.001	1	.976	.008		

A binary logistic regression was used to predict the capacity (preferred role) in which physicians were willing to participate in the long-distance mentorship program. The dichotomous outcome variable of interest was willing to participate either as a mentor or as a mentee. The possible predictors were i) age, ii) the number of years of practice as a public health physician, and iii) year of graduation. The Hosmer and Lame show goodness of fit was not statistically significant (*p* > 0.05 (0.185)), indicating that the model is correctly specified. The -2log likelihood = 102.1, and the Nagelkerke R square = .379. Additionally, about 40% of the variance in the dependent variable is explained by the predictor variable in the model, with percentage accuracy classification, PAC =82.8%.

The binary logistic model resulted in a statistically significant finding for the independent variable (− number of years of practice, B = [−.130], SE = .057, Wald = 5.28, *p* = 0.02). For every unit increase in the number of years of practice, the estimated odds ratio favored a decrease of 12% [Exp (B) = 0.878, 95% CI (.786, .981)] in the likelihood to be a mentee. Therefore, as the number of years of practice increased the odds of being a mentee decreased. The independent variable “age” and “year and graduation” was not statistically significant (*p* = 0.43 and 0.95, respectively).

### Qualitative findings

In-depth interviews were carried out with key informants comprising of college professors and consultants, as shown in Table 7 above. Qualitative findings are presented in four thematic areas: i) organizational capacity to coordinate LDMP; ii) technical expertise and individual competence to provide mentorship; iii) financial capacity to implement and sustain LDMP, and iv) demand for mentorship from potential mentees.

**Table 7:** Characteristics of the respondents

Respondents code	Gender	Rank	Expertise
R1	Male	College professor	Occupational health
R2	Male	College consultant	Communication
R3	Male	College Professor	Environmental health
R4	Female	College consultant	Health management
R5	Male	College Professor	Occupational health
R6	Male	College consultant	Nutrition in Health
R7	Male	College Professor	Health system economics

Table 8 summarizes respondents’ views on requirements for feasibility of LDMP for public health physicians in Nigeria.

**Table 8:** Summary of respondents’ perspectives on requirements for the feasibility of LDMP for public health physicians of Nigeria

Thematic area	Requirements for the feasibility of LDMP for public health physicians
Organizational capacity to coordinate LDMP	➢ A structure that supports postgraduate training (the national and regional colleges) ➢ Secretariat for coordination and M&E ➢ Dedicated administrative unit to manage logistics ➢ Planning committee (technical working group) ➢ Guidelines, standards, and expectations ➢ Physicians at varying stages of career progression
Technical expertise and individual competence to provide mentorship	➢ Skilled, experienced, and willing mentors ➢ Willing and dedicated mentees ➢ Strong ICT support system including hardware and software ➢ Reliable and high-speed internet connectivity (preferably institutional access)
Financial capacity to implement and sustain a LDMP	➢ Availability of funding (adequate and predictable) ➢ Financial commitment expressed through a budget line for a long-distance mentorship program ➢ Staff salaries ➢ Funds for operational cost
Demand for mentorship from potential mentees	➢ Awareness creation about mentorship among members ➢ Good understanding of the benefits of mentorship ➢ Platforms to create awareness ➢ Platforms to enable face-to-face interaction between mentoring pairs

#### Organizational capacity to coordinate a LDMP

There was consensus that the national and regional training colleges (which make up the association) have well-organized structures to support training; therefore, the colleges will be able to champion and administer a long-distance mentorship program. Respondents noted that membership of both colleges comprises categories of people at various stages in their career, and mentorship could easily be incorporated into the training program. However, they highlighted the need for the establishment of a dedicated administrative structure and a planning committee to i) set up logistics for implementation, ii) pilot the affairs of the program, iii) establish guidelines and directives, and iv) monitor and evaluate the program. Some of the respondents noted that the colleges would have to play an active role to properly coordinate all actors and activities of the LDMP. Some respondents further suggested that the existing secretariat could be used for coordinative purposes, while others opined that a new secretariat should be established for effective coordination, monitoring, and evaluation of the program.

*“Yes, the college can* (implement a LDMP)*, but there will be a need for coordination. This could rest on a Chief Programme coordinator, just like we have the Chief examiner. It will need strong coordination, administration, and systems to take action when progress is not being made. An advisory team which will consist of high-level people to provide strategies and guidance for running the program.” (R7, College Professor)*

*There is a need for the college to set up a committee, establish standards and expectations, kick it off, encourage it, facilitate it as much as they can. (R2, College consultant)*

*“The college already has an existing secretariat to work with. It doesn’t need a new one. All it needs to do is to set up a committee to champion it. There is a need for an administrative structure, this will not work with the usual civil service work, we need people who are able to follow things through, in a project sense.” (R2, College consultant)*

Some respondents agreed that the annual national and regional conferences present a very good opportunity to institutionalize mentorship, and should be leveraged upon for the annual face-to-face meetings between mentors and mentees. They suggested that a mentorship session should be included in the conference sessions, specifically for mentoring activities.

*“The already existing conferences can be leveraged upon and time can be set aside, probably a day, for interaction between the mentors and mentees.” (R2, College Consultant)*

Two respondents expressed reservations about the capacity of the training colleges to coordinate a formal LDMP. They felt that coordination of the program should be done at the institutional level and that the role of the college is that of demand creation and establishment of guidelines.

*“They can organize it, to an extent, but not absolutely. They can create awareness and establish guidelines for such mentorship arrangements. They can also enlightenment people to understand the need for it. That is the extent to which the colleges can get involved” (R4, College Professor)*

*“long-distance mentorship is the way to go, it very practical, but the college will not be able to administer and run it, they can only promote it, while individual institutions will be in a better position to administer it” (R5, College Professor)*

#### Technical expertise and individual competence to provide mentorship

There was a consensus that the association has highly skilled and experienced mentors, who are willing to mentor the younger colleagues; and also, willing and dedicated mentees who are eager to be mentored. However, respondents highlighted the need for a strong Information and Communications Technology (ICT) system to be set up to manage technicalities and ensure the smooth running of the program. Being a long-distant mentorship program, it will require a lot of virtual communication between participants, it will also require virtual monitoring.

Respondents further highlighted the need for a system that will enable and monitor communication, as well as provide support to ensure meaningful engagement between participants. They also noted the need for other technical equipment and facilities: software applications for audio-visual communication, reliable internet connectivity, laptops, pads, phones. They agreed that reliable and efficient internet is far-fetched in most institutions, highlighting that college members mostly depend on their private internet, even when there is institutional access. This is due to the unreliable nature of institutional internet, even when available. Respondents however noted that non-governmental organizations have more reliable internet access than most government-owned institutions. They stressed the need for the provision of efficient and reliable institutional internet access across all institutions under the colleges

*“We have a lot of highly skilled personnel in various fields of expertise. The college has vast human resources that are underutilized, and this is one of the requirements for such a program. However, it requires a very strong ICT system to be set up, to be able to administer and coordinate the program. Also, support staff to handle the technical issues as they arise. The existing secretariat might suffix for cost efficiency. But there is need for identification of ICT experts” (R6 College Consultant)*

*A technical working group and a strong secretariat will be needed. It requires people who are trained to a tracking system for proper coordination and feedback. It will also need an advisory team which will consist of high-level people to provide strategies and guidance for running the program.” (R7 College Professor)*

#### Financial capacity to implement and sustain a LDMP

Some of the respondents highlighted that implementing a long-distance mentorship program requires a substantial financial commitment. They stressed that the colleges must be able to make meaningful commitments to start and sustain such a program. They must create a budget line for mentorship and ensure that funds are released for operational costs. There was consensus that the colleges may not be able to implement LDMP given their current financial capacities and the funding gaps that exist for other activities.

*“There are financial gaps because it will definitely require some finance. The college needs to identify the importance of the mentorship program and the future benefit. If they can think futuristic, they should be able to create a budget line for mentorship from the money they are already getting to be able to institute such a program.” (R6 College Consultant)*

***“****Money cuts across everything and will also be needed for payment of staff and running the secretariat.” (R2, College Consultant)*

#### Demand for mentorship from potential mentees

Demand creation was a recurrent theme amongst the respondents. Respondents agreed that there is a poor understanding of the concept of mentorship and its relevance among members of the association, and this may result in poor demand for mentorship from potential mentees. Creating awareness to enable college members to understand the benefits of mentorship was recommended as a strategy that will enable participation. Some respondents stressed that the most crucial role of the college should be awareness creation, leveraging every opportunity and platform like the annual scientific conference.

*“There is poor awareness and lack of willingness as a result of poor understanding of the concept and the need for mentorship. Willingness on paper is quite different from action [active participation]. There is need for awareness creation to reorient members towards the culture of mentorship” (R4 CollegePprofessor)*

*“The biggest thing is the willingness of members to commit time to do this. There is a need for awareness creation at every level, during conferences at the national and regional level. This will help to get people involved and committed to making it work. The role of the college is to create awareness. (R3, College Professor)*

To sustain the demand for mentorship, respondents opined that the annual national and regional scientific conferences held by both colleges and APHPN present a good opportunity to institutionalize mentorship. These platforms should be leveraged upon to increase uptake and sustain ofthe mentorship program. Respondents suggested that mentoring sessions should be included in the program schedules to enable face-to-face interactions between mentors and their mentees.

*“The already existing conferences can be leveraged upon, and time can be set aside, probably a day, for interaction between the mentors and mentees.” (R2, College Consultant)*

## Discussion

Increasing limitations to one-on-one mentorship due to conflicting schedules and geographic separation continue to deter mentorship, a very important aspect of career progression. Long-distance mentorship bridges this gap, but this has not been explored amongst public health physicians. Evidence on the feasibility of establishing a formal long-distant mentorship program for public health physicians of Nigeria may set the implementation processes into motion. We used in-depth interviews to explore the feasibility and used a cross-sectional survey to ascertain the willingness of public health physicians to participate in a formal long-distance mentorship program.

Public health physicians are willing to participate in a long-distance mentorship program. This was highly expected considering that the period of residency in public health represents a critical training phase, during which the physician is expected to acquire practical skills and expertise for sound public health practice. The field of public health is such that needs guidance and coaching for goal-oriented navigation [18]. Mentoring in this phase of career development creates an enabling environment for trainees to internalize skills for interpretation of public health realities, research, and population health interventions [19]. It is not surprising that academic and research-oriented public health physicians are willing to mentor and be mentored. However, qualitative findings showed that the high percentage of willingness seen in the survey may not necessarily equate to practical willingness. This further buttresses the need for intense awareness creation at all levels to get members of the association on board.

In-depth interviews highlighted various elements of feasibility required to implement a LDMP program: organizational, technical, financial, and market feasibility. These elements are critical for feasibility, as they were captured by a framework proposed by Mbuagbaw et al. for setting up a long-distance mentorship program for researchers. The authors developed the framework through a comprehensive literature review and an appraisal of the first Canadian Institutes of Health Research - Canadian HIV Trials Network international postdoctoral fellowship program. They identified the components of a long-distance mentorship program, categorizing them into three classes- critical, important, and supportive components. This framework aligns with the components of feasibility identified in our findings. It captured “technical expertise” as a critical component, while it captured “coordination” and “funding” as important components for planning a long-distance mentorship program [4].

The framework also captured the “enabling environment” as critical for setting up a long-distance mentorship program. This is a crucial requirement, as successful mentorship in academic medicine requires an enabling academic environment for effective mentorship [20]. The colleges provide such enabling environment - both the National Postgraduate Medical College of Nigeria and West African College of Physicians are well-organized institutions that can incorporate a formal organized long-distance mentorship program for the APHPN as a whole. However, there is a need for proper coordination of the process. All successful mentoring programs require intensive coordination; mechanisms for recruitment; criteria for matching partners; strategies for providing support during mentoring relationship development; documentation of progress and challenges; and evaluation of the results [21].

Stakeholders acquiesced to the need for proper coordination of the program, such that will involve a mentorship committee, a technical working group, a strong ICT team, and other administrative staff for effective program implementation. However, stakeholders differed on the level of coordination; the majority proposed central coordination (at the college level), while the minority opined a decentralized coordination (at the level of various institutions under the college). Coordinating at the institutional level will limit preferred mentor-mentee pairing; a mentee may not be able to find a desirable mentor within his or her institution with respect to the desired areas of expertise and other considerations. The college has diverse experts dispersed all over member institutions; college members in a particular institution may not be aware of the human resources or potential mentors available in other institutions under the college.

Therefore, the machinery of the college and the association can be leveraged to create coordinated linkages between mentoring pairs. A well-structured mentoring program that connects early-career researchers with diverse experts may stimulate new networking possibilities and lead to effective collaborations amongst investigators with different skills and expertise [22]. Furthermore, mentoring networks that leverage diverse virtual methods to connect multiple institutions have great potential to foster organizational cultures that will sustain quality mentorship in research [23].

We identified enabling factors that have great potential to foster and sustain collaborations and quality mentorship amongst public health physicians- the vast wealth of highly skilled and well-experienced mentors and dedicated mentees who are willing to participate in the program; the training structure of the residency program, which creates an enabling environment for incorporating a mentorship program within the colleges; the already existing annual national and regional conferences that could also be leveraged upon for demand creation to reinforce the process. The annual conferences could also be an avenue for an initial face-to-face meeting for mentoring pairs. However, this may require an additional day to the usual conference period, for more effective and meaningful face-to-face interaction between mentoring pairs. Face-to-face interaction that precedes subsequent virtual interactions has been found to foster adequate interaction between mentoring pairs, enabling more meaningful relationships.

Virtual communication is a fundamental requirement for long-distance mentorship [24]. Our findings showed that public health physicians who are willing to participate in the mentorship program were also willing to engage in virtual meetings for communication purposes. The mentoring framework describe by Mbuagbaw et al. also captured communication as a critical component of LDMP. Virtual meetings and conferencing have become a global phenomenon, offering opportunities for communication, dissemination, and sharing of information for different intents and purposes without geographic barriers [25]. Institutions and organizations are making use of virtual opportunities in diverse ways [26]. The COVID − 19 pandemic has also accelerated virtual processes across the globe. Our inquiries highlighted reliable internet connectivity as an essential requirement for easy connectivity, but still lacking at institutional levels. Setting up fast and reliable internet networks across institutions will facilitate swift online conferencing and virtual mentorship meetings, even during office hours. Virtual meetings and conferences are not just useful and suitable for mentorship, they also save time and travel cost [27, 28].

Based on our findings, public health physicians of Nigeria should pay close attention to starting a formal LDMP that will enable upcoming physicians to receive good mentorship, and also build their capacity to become good mentors as well. This is critical for quality training and sustaining institutional excellence. It is not surprising that the number of years that a physician has practiced public health can predict the capacity in which the respondent will participate in the mentorship program (either as a mentor or as a mentee). The likelihood of becoming a mentor increased with every unit rise in the number of years of practice, further buttressing the need for the mentoring process to commence as soon as trainees gain entrance into the program. This is crucial because the mentoring process does not only prepare trainees for excellent public health practice but also prepares them to become a mentor to younger and upcoming physicians. This form of continuity will inculcate the culture of mentorship in the association, and foster excellence and successful career progression amongst public health physicians of Nigeria.

### Strengths and weaknesses of the study

The convergent model of mixed-method enabled triangulation and integration of findings for more robust syntheses. In-depth interviews also enabled deeper exploration of stakeholders’ perspectives on the various components of feasibility, and gaps that must be filled to be able to implement a long-distance mentorship program amongst public health physicians of Nigeria. However, in-depth interviews did not ensure maximum variability and representation across the length of years of practice, as residents were not captured in the interviews. Also, the quantitative survey was limited to only members of APHPN who attended a particular scientific conference. Therefore, generalization of findings may not be made with certainty for members who did not attend the conference.

## Conclusion

Public health physicians in Nigeria are willing to participate in a formal long-distance mentorship program. There is a great opportunity to leverage the organizational structure of the training colleges for program implementation. The existing vast human resources, training structure of the college, enabling academic environment, and annual conferences that assemble members annually are factors that will enable the process. However, there are identified gaps that must be put in place for a successful program- robust coordination at the college level, a good financial plan, and strong Information and Communications Technology (ICT) system are needed to ensure the smooth running of the program. Significant program uptake will require personal commitment and intense demand creation, both institutional and college level. Given the relevance of mentorship for successful career progression and the limitations of the traditional face-to-face method of mentoring, instituting a formal mentorship program should be considered for the public health physicians of Nigeria. It is, therefore, crucial to explore contextual strategies and considerations for initiating and implementing formal long-distance mentorship program, with peculiarities to public health physicians of Nigeria.

## Data Availability

The datasets used and/or analyzed during the current study are available from the corresponding author on reasonable request.

## Abbreviations

APHPH: Association of Public Health Physicians of Nigeria
FCT: Federal Capital Territory
LDMP: Long-Distance Mentorship Program
NPMCN: National Postgraduate Medical College of Nigeria
PHPN: Public Health Physicians of Nigeria
WACP: West African College of Physicians
